# The *Mytilus chilensis* Steamer-like Element-1 Retrotransposon Antisense mRNA Harbors an Internal Ribosome Entry Site That Is Modulated by hnRNPK

**DOI:** 10.3390/v16030403

**Published:** 2024-03-05

**Authors:** Leandro Fernández-García, Constanza Ahumada-Marchant, Pablo Lobos-Ávila, Bastián Brauer, Fernando J. Bustos, Gloria Arriagada

**Affiliations:** Instituto de Ciencias Biomedicas, Facultad de Medicina y Facultad de Ciencias de la Vida, Universidad Andres Bello, Santiago 83700071, Chile; leandrofg1990@gmail.com (L.F.-G.); ahumadamarchantc@gmail.com (C.A.-M.); p.lobosvila@uandresbello.edu (P.L.-Á.); babrauerc@gmail.com (B.B.); fernando.bustos@unab.cl (F.J.B.)

**Keywords:** retrotransposon, translation initiation, IRES, hnRNPK, ITAF

## Abstract

LTR-retrotransposons are transposable elements characterized by the presence of long terminal repeats (LTRs) directly flanking an internal coding region. They share genome organization and replication strategies with retroviruses. Steamer-like Element-1 (*Mch*SLE-1) is an LTR-retrotransposon identified in the genome of the Chilean blue mussel *Mytilus chilensis*. *Mch*SLE-1 is transcribed; however, whether its RNA is also translated and the mechanism underlying such translation remain to be elucidated. Here, we characterize the *Mch*SLE-1 translation mechanism. We found that the *Mch*SLE-1 5′ and 3′LTRs command transcription of sense and antisense RNAs, respectively. Using luciferase reporters commanded by the untranslated regions (UTRs) of *Mch*SLE-1, we found that in vitro 5′UTR sense is unable to initiate translation, whereas the antisense 5′UTR initiates translation even when the eIF4E-eIF4G interaction was disrupted, suggesting the presence of an internal ribosomal entry site (IRES). The antisense 5′UTR IRES activity was tested using bicistronic reporters. The antisense 5′UTR has IRES activity only when the mRNA is transcribed in the nucleus, suggesting that nuclear RNA-binding proteins are required to modulate its activity. Indeed, heterogeneous nuclear ribonucleoprotein K (hnRNPK) was identified as an IRES trans-acting factor (ITAF) of the *Mch*SLE-1 IRES. To our knowledge, this is the first report describing an IRES in an antisense mRNA derived from a mussel LTR-retrotransposon.

## 1. Introduction

LTR-retrotransposons (LTR-RTs) represent the most active type of genetic mobile elements in animal genomes [1,2]. LTR-RTs and retroviruses share a common evolutionary ancestry and genetic structure, both having an internal coding region flanked by two long terminal repeat (LTR) sequences [3,4]. Both categories of elements encode *gag* and *pol* genes, and LTR-RTs usually lack or have interrupted the *env* gene that allows for retroviruses to move between cells [2,5]. Retroviral and LTR-RT mRNAs are synthesized by RNA polymerase II; therefore, as the vast majority of cellular mRNAs, they are modified in the 5′ with a cap and in the 3′ with a poly(A) tail [6,7]. Additionally, some retroviruses such as Human Immunodeficiency Virus type 1 (HIV-1) and Human T-lymphotropic virus type 1 (HTLV-1) as well as the retrotransposon Long Interspersed Element-1 (LINE-1) generate mRNAs through antisense transcription [8,9,10,11], mediated by the promoter activity in their 3′LTR [12,13].

Little is known about the translation mechanisms employed by retrotransposon mRNAs. Nevertheless, considering the similarity of retrotransposon RNAs to retroviral RNAs, it is expected that both elements use similar mechanisms to ensure protein synthesis. It is known that retroviral mRNA translation occurs mainly by two mechanisms: the canonical cap-dependent translation and the internal ribosome entry site (IRES)-mediated translation [14]. The canonical or cap-dependent translation mechanism relies on the recognition of the 5′ cap structure, and the recruitment of the translation machinery is dependent on this event [15]. This process begins with the recognition of the RNA 5′ cap structure by the eukaryotic initiation factor 4 E (eIF4E) [16]. eIF4E is part of the eukaryotic initiation complex F (eIF4F), formed additionally by the ATP-dependent RNA helicase eIF4A, and the scaffolding protein eIF4G. Recruitment of the 40S ribosomal subunit to the mRNA is mediated by the eIF4E-eIF4G interaction [17]. eIF4G interacts with eIF3, which is pre-bound to the multifactor 43S complex that includes eIF2, eIF1, eIF1A, eIF5 and the Met-tRNAi^Met^ [17]. Translation initiation is also regulated by the 5′ cap-3′ poly(A) interaction that synergistically stimulates mRNA translation efficiency in a closed circular conformation involving the eIF4E-eIF4G–poly(A)-binding protein (PABP) interaction [16,18]. Once the 40S subunit is recruited, it scans the mRNA in the 5′ to 3′ direction until it identifies the start codon in an optimal context GCC(A/G)CCAUGG, with a purine at the –3 and a G at the +4 positions [19]. After the translation initiation site has been identified, the assembly of a competent ribosome (80S) occurs and peptide synthesis begins [16]. This mechanism is the main strategy used by cellular mRNAs to initiate translation.

On the other hand, IRESs are RNA regions with the ability to recruit the translation machinery independently of the 5′ cap and can be located in the UTR or in the open reading frame (ORF) [14,20]. IRESs were discovered in 1988 in the *Picornaviridae* virus family, whose mRNA lacks a 5′ cap [21,22]. Afterward, diverse types of IRESs have been identified in 5′-capped viral and cellular mRNAs [23]. Among the IRES diversity, there are differences in the mechanism to recruit the 40S subunit (scanning-dependent or direct recruitment to the initiation codon) and on the assistance of eIFs to initiate translation [20]. In addition to eIFs and the 40S, some IRESs require host RNA-binding proteins (RBPs) to be functional. These RBPs regulate the IRES activity and are named IRES trans-acting factors (ITAFs) [24]. ITAFs modulate IRES activity through RNA–protein and/or protein–protein interactions forming a ribonucleoprotein complex, the IRESome, which has been mostly studied in picornaviral [25] and retroviral IRESs [14].

Retroviral IRESs have the function to ensure the synthesis of viral proteins when canonical translation is inhibited by the host response or by the virus for their own benefit [26]. The unspliced RNAs of HIV-1, HIV-2, Mouse mammary tumor virus (MMTV), Simian immunodeficiency virus (SIV), Feline leukemia virus (FLV) and HTLV-1 harbor IRESs [14]. An intriguing case is HTLV-1 where both full-length sense and the spliced antisense mRNAs exhibit IRES activity [27,28]. In the case of retroelements, there is limited information available. To date, it is known that the messengers of LINE-1 [29], *Drosophila* gypsy [30] and Virus-like 30 (VL30) [31] harbor 5′UTR with IRES activity.

Steamer is an LTR-RT present in the genome of the soft-shell clam *Mya arenaria*, whose expression and copy number are associated with transmissible cancer in animals [5,32,33]. Several Steamer-like LTR-RTs have been described in mollusks [34,35]; among them, Steamer-like element-1 (*Mch*SLE-1) was recently described in the genome of the Chilean blue Mussel *Mytilus chilensis* [36]. A genetic analysis of the *Mch*SLE-1 sequence reveals that the positive and negative DNA strands contain intact ORFs. This observation suggests that this LTR-RT could generate sense and antisense RNAs. In this study, we investigate whether both sense and antisense RNAs are indeed produced in vivo. Furthermore, we characterize the translation initiation mechanism mediated by *Mch*SLE-1 5′UTRs. Our findings describe for the first time evidence of an IRES in an antisense RNA within a long terminal repeat retrotransposon in mollusks.

## 2. Materials and Methods

### 2.1. Plasmids

The plasmids dl HIV-1 IRES, dl HCV IRES, dl ∆EMCV, Glo-FLuc, pFMDV-Leader Protease, pmusHBZ FLuc and pCMV-HA-hnRNPK were kindly provided by Dr. Marcelo López-Lastra (Pontificia Universidad Católica de Chile) and have been previously described [28,37,38,39,40,41]. To generate the SLE-1 dual-luciferase (dl) bicistronic vector, dl SLE-1 5′UTR sense, the SLE-1 5′UTR sense was amplified by PCR from the *M. chilensis* genome, using the primers SLE1_5UTR_S_EcoRI_F (5′AAATTTGAATTCACAATGTAGACTAACCATGCTG 3′) and SLE1_5UTR_S_AUG1_NcoI_R (5′ AAATTTCCATGGCGGGTTTTAACATCTGACACC 3′), designed using the *Mch*SLE-1 sequence deposited at NCBI (OR712106). To generate the dl SLE-1 5′UTR antisense plasmid, the SLE1_5UTR_AS_EcoRI_F (5′AAATTTGAATTCGTTGGGCGGAAGTGCCAGTGCGCAT 3′) and SLE1_5UTR_AS_AUG1_NcoI_R (5′ AAATTTCCATGGGATGAAAACAAAAACAATAGTTCG 3′) primers were used. The amplicons were digested using *EcoR*I and *Nco*I and were cloned into the intercistronic space of the dl HIV IRES, digested previously with *EcoR*I/*Nco*I and *Nco*I/*Xba*I as described in [40]. The promoterless vector ∆SV40 dl SLE-1 5′UTR antisense was generated by digesting the dl SLE-1 5′UTR antisense plasmid with *Mlu*I and *Stu*I, followed by Klenow fragment treatment and ligation with T4 DNA Ligase. The monocistronic mSLE-1 sense and mSLE-1 antisense plasmids were generated by enzymatic digestion of dl SLE-1 5′UTR sense and dl SLE-1 5′UTR antisense with *Stu*I and *EcoR*I, followed by Klenow fragment treatment and ligation with T4 DNA Ligase. To generate the plasmid pAS-SLE-1-GFP, the AS SLE-1 ORF coding was amplified from *M. chilensis* genomic DNA using the primers AS_ORF_NheI-F (5′ AAATTTGCTAGCATGAGATATCTCTTGTTCATT 3′) and AS_ORF_nostop_HindII-R (5′ AAATTTAAGCTTACACCACAAGATCGACAAAAA 3′). The PCR product was digested using *Nhe*I and *Hind*III and ligated into pEGFP-N1 (Clontech) digested with the same restriction enzymes. All the constructed vectors were verified by sequencing and are available upon request.

### 2.2. Cell Culture and Nuclear Extract Preparation

HeLa cells (ATCC-CCL-2) were grown at 37 °C with 5% CO_2_ in Dulbecco’s modified Eagle medium (DMEM) supplemented with 10% fetal bovine serum, 100 IU/mL penicillin and 100 µg/mL streptomycin.

To prepare the nuclear extracts, confluent HeLa cells were washed twice with cold phosphate saline buffer (PBS) containing Pierce™ Protease Inhibitor (protease inhibitors). The cells were mechanically detached by scraping and centrifuged at 1000× *g* for 5 min at 4 °C. The cell pellet was resuspended in buffer A (50 mM Hepes pH 7.3, 3 mM Mg_2_Cl, 20 mM KCl, 1% NP40, 1 mM DTT and protease inhibitors) and incubated for 10 min at 4 °C, followed by mechanical disruption by 20 dounce strokes. After centrifugation at 3500× *g* for 15 min, the nuclear pellet was resuspended in one volume of buffer B (10 mM Hepes pH 7.0, 300 mM NaCl, 1.5 mM MgCl_2_, 0.2 mM EDTA, 10% Glycerol, 1 mM DTT and protease inhibitors) and incubated for 1 h at 4 °C and then passed 20 times through a 25 gauge syringe and finally centrifuged at 13,800× *g* for 15 min at 4 °C. The supernatant was recovered, and the protein content was quantified. Working dilutions at 250 and 500 ng were prepared using buffer C (25 mM Hepes pH 7.3, 2 mM MgCl_2_, 50 mM KCl and 75 mM KAc) and stored at −80 °C until use.

### 2.3. Mussels RNAs and DNA Samples

The RNA isolated from the hemocytes of 11 mussels were treated with TURBO™ DNase (Ambion, Thermo Scientific, Waltham, MA, USA) and precipitated with LiCl before specific cDNA synthesis and quantitative PCR (qPCR). Genomic DNA was also isolated from the *M. chilensis* hemocyte as described in [5].

### 2.4. cDNA Synthesis and Quantitative PCR

For strand-specific cDNA synthesis, M-MuLV Reverse Transcriptase (New England Bio Labs, Ipswich, MA, USA) was used according to the manufacturer′s instructions. A total of 100 ng of RNA was incubated with 10 µM dNTP, SLE1F_qPCR (5′-CAGACGAGAGCGATAGCGAA-3′) for the sense strand or SLE1R_qPCR (5′-GCAGTCGCACCGCTATCTAA-3′) for the antisense strand or MchTub-Rv (5′-TGGACGAAAGCACGTTTGGC-3′) primer as housekeeping. 

To obtain the cDNA from the rabbit reticulocyte lysates (RRLs), the samples were diluted 1:100 in nuclease-free water and 10 µL of this dilution was used for cDNA synthesis. cDNA synthesis was performed using an iScript gDNA clear cDNA Synthesis Kit (Biorad) according to the manufacturer′s instructions. qPCR experiments were carried out using Brilliant II SYBR Green QPCR Master Mix (Agilent Technologies, Santa Clara, CA, USA). FLuc RNA was detected with firefly sense (5′-ACTTCGAAATGTCCGTTCGG-3′) and antisense (5′-GCAACTCCGATAAATAACGCG-3′) primers, while 18S RNA was detected using 18S sense (5′-GTGGAGCGATTTGTCTGGTT-3′) and 18S antisense (5′-CGCTGAGCCAGTCAGTGTAG-3′) primers. For strand-specific qPCR, the primers SLE1F_qPCR and SLE1R_qPCR described above and *Mch*Tub-Fw (5′-GAGCCGTCTG-CATGTTGAGC-3′) and *Mch*Tub-Rv (5′-TGGACGAAAGCACGTTTGGC-3′) were used. The data were analyzed using the ΔΔCT method [42,43].

### 2.5. In Vitro Transcription

In vitro transcribed and capped mRNAs were generated using mMESSAGE mMACHINE™ (Ambion, Thermo Scientific), followed by the addition of a poly(A) tail using a Poly(A) Tailing Kit (Ambion, Thermo Scientific). The mRNAs with an unfunctional cap (Acap) were synthesized using mMESSAGE mMACHINE but substituting the capping buffers with a mix of rNTP and 40 mM A(5′)ppp(5′)A cap analog (NU-506-5, Jena Bioscience, Jena, Thuringia, Germany). The uncapped RNAs were synthesized in the absence of any cap analog. The T7 promoters DNA templates of the SLE-1 (S and AS) reporters were generated by PCR using SLE1 5UTR_S_T7 (5′-TAATAC-GACTCACTATAGACAATGTAGACTAACCATGCTGTA-3′) or SLE1 5UTR_AS_T7 (5′-TAATACGACTCACTATAGGTTGGGCGGAAGTGCCAGTGCGCAT-3′) and the FLuc_R_Xba_Xho3stop (5′-AAATTCTCGAGTCTAGACTATCATTACAGGCGATCTTTCCGCCCTTCTTGGCC-3′). The globin template was generated by enzymatic digestion of the GloFLuc plasmid with *EcoR*I as previously described [27,40]. The plasmid-encoding foot-and-mouth disease virus leader (FMDV L) protease was digested with *Xba*I. Bicistronic mRNA reporter templates were generated by enzymatic digestion of each indicated plasmid using *BamH*I. All the DNA templates used for in vitro transcription were purified by precipitation with 1/10th volume of 5 M NH_4_OAc and 2 volumes ethanol. After transcription, the reactions were treated with TURBO™ DNase (Ambion, Thermo Scientific) to eliminate the templates. The mRNA was precipitated with 2.5 M LiCl, RNA concentrations were determined spectrophotometrically and the RNA integrity was monitored by electrophoresis on denaturing agarose gels.

### 2.6. In Vitro Translation

In vitro translation experiments were performed using the cell-free system nuclease-treated RRL (Promega Corporation) in the presence of standard salt concentrations (79 mM Kac and 0.5 mM MgAc). The translation of mGlo, mSense SLE-1 and mAntisense SLE-1 mRNAs was performed using 1 ng of RNA per reaction on 50% (*v*/*v*) RRL at 30 °C for 90 min. For reactions with the cap-dependent translation inhibited by FMDV L protease, the FMDV L protease mRNA was synthesized and translated in vitro as described above and in [40]. The L-protease produced in RRL was diluted in nuclease-free water, and 2 or 6% (*v*/*v*) was added to the new RRL and incubated for 15 min at 30 °C before the mGlo and mAntisense SLE-1 mRNAs were translated as described above.

To determine the influence of the nuclear extracts on the *Mch*SLE-1 IRES activity in vitro, 10 ng of the in vitro transcribed mRNA was incubated with 1 μg of HeLa cells nuclear extracts or the equivalent volume of buffer C for 10 min. RRL was added and translation was carried out as described above. 

### 2.7. DNA, RNA and siRNA Transfection

HeLa cells were seeded in 48- or 24-well plates at 30,000 or 60,000 cells per well, respectively. Twenty-four hours later, the cells were transfected using polyethyleneimine (PEI). Each well was transfected with 200 ng of DNA corresponding to the dual-luciferase reporter indicated in each figure or co-transfected with the indicated amount of pCMV-HA-hnRNPK plasmid. In all cases, the pSP64 poly(A) plasmid was used to maintain the same amount of DNA in all transfection conditions.

For the RLuc silencing experiments, the HeLa cells seeded in 24-well plates were co-transfected with dl SLE-1 5′UTR AS and 50, 100 or 200 nM of the siRLuc RNA (5′-UAUAAGAACCAUUACCAGAUUUGCCUG-3′) using Lipofectamine 2000, and the luciferase activity was measured 24 h post transfection.

For endogenous hnRNPK silencing, the HeLa cells seeded in 24-well plates were co-transfected using Lipofectamine 2000, with 200 ng of dl SLE-1 5′UTR AS and 20 nM of Dicer-Substrate siRNA targeting the 3′UTR of the hnRNPK mRNA [41] or a scrambled siRNA as the negative control. Both siRNAs were kindly provided by Dr. Marcelo López-Lastra (Pontificia Universidad Católica de Chile).

### 2.8. Luciferase Assays

Twenty-four hours post transfection, the cells were lysed using 50 μL of passive lysis buffer (PLB) (Promega corporation) and the FLuc and RLuc activities were measured using 20 µL of cell lysates and 20 µL of the dual-luciferase reporter (DLR) assay system (Promega Corporation) using a Synergy H1 Multimode Reader (Agilent).

### 2.9. Western Blot

To detect the eIF4G proteolytic cleavage mediated by the FMDV L protease in RRL, the RRL reaction was resuspended in 100 µL of PLB. To detect the overexpression of HA-hnRNPK and to confirm the silencing of hnRNPK, the same cell lysate used to measure the luciferase activity was used for protein detection. To detect the expression of the AS-SLE-1-GFP fusion protein, cells transfected with different amounts of the coding plasmid were lysed in PLB 24 h post transfection. In all cases, 40 µg of the whole protein lysate was boiled in 5X sodium dodecyl sulfate (SDS) loading buffer, and the proteins were resolved in 10% SDS-PAGE and transferred to PVDF membranes. After blocking with 5% of milk in PBS, the membranes were incubated with the primary antibodies eIF4G (Santa Cruz 13315S, 1:1000), eIF4E (Santa Cruz 271480, 1:3000), HA-epitope (Cell Signaling 3724S, 1:2000), hnRNPK (Santa Cruz 28380, 1:5000), GFP (Santa Cruz 9996, 1:1000) or GAPDH (Cell Signaling 2118SS, 1:5000). Secondary HRP-conjugated anti-mouse or anti-rabbit antibodies were used. The Western blots were visualized by a chemiluminescence reaction with SuperSignal West Pico (Thermofisher, Waltham, MA USA).

### 2.10. Statistical Analysis

The values are presented as the mean ± standard error of the mean (SEM) for 3 or more independent experiments All the statistical analyses were performed using Graphpad Prism (GraphPad Software Inc., San Diego, CA, USA) with a *t* test or one-way ANOVA with multiple comparisons using the Tukey test. Values of *p* < 0.05 were considered statistically significant.

## 3. Results

### 3.1. MchSLE-1 RNA Is Transcribed from Both DNA Strands

**Figure 1:**
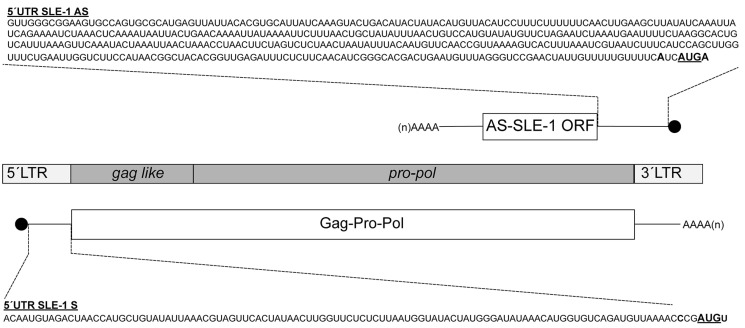
Genomic structure of *Mch*SLE-1 retrotransposon and their two putative transcripts. Steamer-like Element 1 (*Mch*SLE-1) contains in the sense strand a complete open reading frame (ORF) of 1376 amino acids (gag and pro-pol), flanked by LTR regions, where the 5′LTR could command transcription of the sense mRNA (*Mch*SLE-1 S). On the antisense strand, *Mch*SLE-1 contains an ORF of 67 amino acids with an AUG codon in a KOZAK context, named Antisense SLE-1 ORF (AS-SLE-1). The 3′LTR could act as a promoter for the transcription of an antisense mRNA (*Mch*SLE-1 AS). The sequences of the S and AS 5′UTRs used in this article are shown. The translation initiation codon of each ORF is depicted in bold letters and underlined. Note that the scheme is not to scale.

*Mch*SLE-1 is a novel LTR-RT (4.76 Kbp) recently described in the genome of *M. chilensis* [36]. From the sense strand, we identified a single ORF encoding a polyprotein of 1376 amino acids where the most conserved residues correspond to the retroviral Pol regions, including similarities with the retroviral protease with a diagnosed DSG active site motif [44], a reverse transcriptase (RT) with a polymerase domain containing a YxDD box [45], an RNAse H domain with a diagnostic DEDD catalytic core [46] and an integrase (IN) with an HHCC zinc finger and a conserved D,D(35)E motif [47] (Figure 1). In the amino terminal of the polyprotein, the only Gag similarity is a nucleocapsid domain with two putative zinc fingers containing CCCC and CCHC motifs. A sequence analysis of *Mch*SLE-1 DNA revealed that the positive-strand ORF has an initiation codon in a weak context, while the negative strand encoding a putative protein of 67 amino acids has an initiation codon in the KOZAK consensus. Both DNA strands have poly-adenylation signals, suggesting that both mRNAs could allow for protein translation (Figure 1).

**Figure 2:**
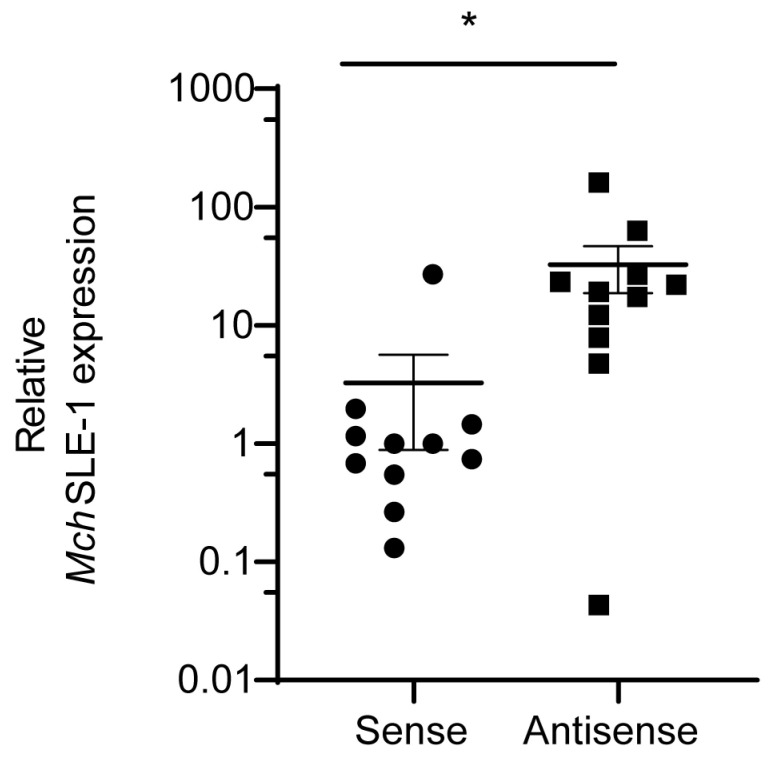
*Mch*SLE-1 is transcribed as two RNAs, sense (S) and antisense (AS). RNA from 11 *M. chilensis* mussels was obtained, strand-specific cDNA was generated and qPCRs specific for *Mch*SLE-1 and *M. Chilensis* tubulin were performed. The relative expression of each transcript to the housekeeping tubulin is shown. Each RT-qPCR was conducted in duplicate. A statistical analysis was performed using *t* tests, * = *p* < 0.05.

First, we verified the expression of the *Mch*SLE-1 sense (S) and antisense (AS) mRNA in animals using strand-specific reverse transcription and qPCR (RT-qPCR). These experiments confirmed that both strands were transcribed in all the analyzed animals (Figure 2). Interestingly, the *Mch*SLE-1 AS RNA was significantly more expressed than the *Mch*SLE-1 S RNA (Figure 2). These results suggest that both the 5′and 3′LTRs of *Mch*SLE-1 could function as promoters for transcription.

### 3.2. MchSLE-1 5′ UTR AS Initiate Translation in a Cap-independent Manner In Vitro

**Figure 3:**
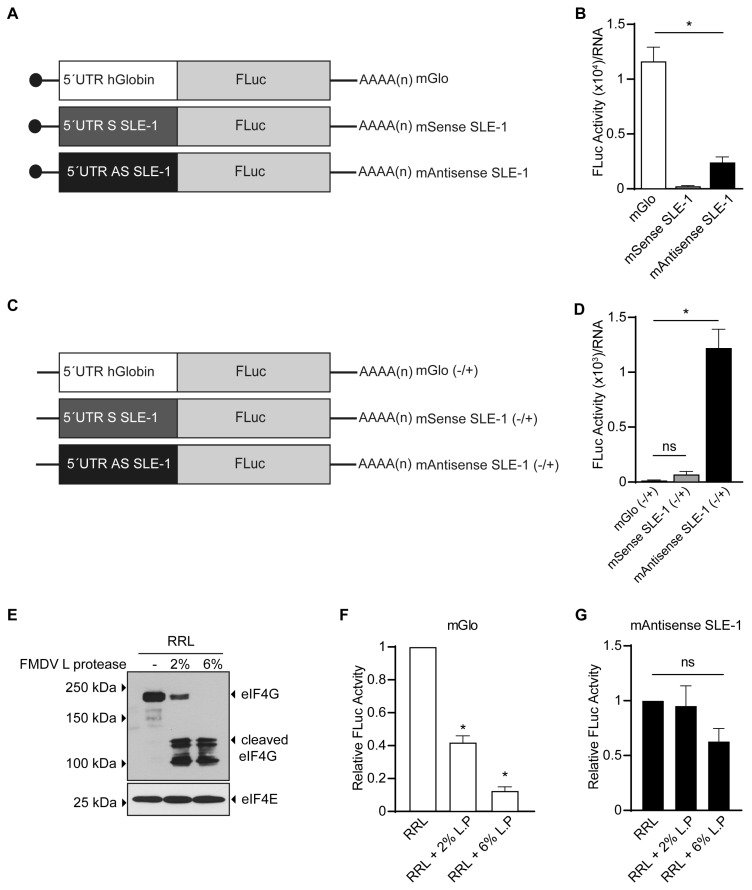
*Mch*SLE-1 5′ UTR AS initiates translation in a cap-independent manner in vitro. The translation commanded by the S and AS 5′UTR of *Mch*SLE-1 was tested in vitro using RRL. (**A**) A schematic representation of the reporter mRNAs evaluated. The black dots represent the 5′cap structure. (**B**) The translation activity of the mGlo, mSense SLE-1 and mAntisense SLE-1 reporters was assayed in vitro using RRL, and the luciferases activity was measured and normalized by the RNA quantity measured by RT-qPCR. (**C**) A schematic representation of the uncapped reporter mRNAs evaluated. (**D**) The translation activity of the uncapped mGlo, mSense SLE-1 and mAntisense SLE-1 reporters was assayed in vitro using RRL, and the luciferases activities were measured and normalized by the RNA quantity measured by RT-qPCR. (**E**) A Western blot to verify the proteolytic cleavage of eIF4G by FMDV L protease. The migration of intact or cleaved eIF4G, and the loading control eIF4E are depicted on the right-hand side, and the migration of the molecular markers is depicted on the left-hand side. (**F**) The translation activity of mGlo in RRL treated with FMDV L protease was assayed. (**G**) The translation activity of mAntisense SLE-1 in the presence of FMDV L protease was tested. In (**F**,**G**), the results are presented as relative to the untreated translation reaction. The values are the means of three independent experiments each performed in triplicate. A statistical analysis was performed by a one-way ANOVA, followed by a Tukey′s multiple test comparison. * = *p* < 0.05, and ns = not significant.

Since *Mch*SLE-1 is transcribed as two RNAs from the S and AS strands (Figure 2), and both strands contain an ORF, we evaluated if these RNAs could function as mRNAs. For this, we generated monocistronic firefly luciferase (FLuc) reporters, where the *Mch*SLE-1 5′UTR S or *Mch*SLE-1 5′UTR AS (depicted in Figure 1) are commanding translation. These constructs were named mSense SLE-1 and mAntisense SLE-1 (Figure 3A). As controls for cap-dependent translation initiation, we used the mGlo reporter where the 5′UTR of human globin mRNA commands FLuc synthesis [27,40] (Figure 3A). After in vitro transcription and translation, the luciferase activity was measured. In this system, the mSense 5′UTR was unable to command the translation initiation, while the mAntisense 5′UTR-driven translation can be detected, although it was significantly lower than the control (Figure 3B). These results suggest that the mSense 5′UTR is insufficient to initiate translation in vitro, while the mAtisense 5′UTR can mediate the translation initiation in RRL. We also assayed the capacity of both 5′UTRs to command translation in the cell culture. For this, we delivered plasmids that generate the same mRNAs used in vitro, with the difference that the control of the cap-dependent translation initiation was commanded by the 5′UTR of the unspliced HBZ, one of the antisense mRNAs of HTLV-1 [27] (Supplementary Figure S1A). We observed that although both mSense 5′UTR and mAntisense 5′UTR reporters have significantly lower activity than the control, this time both had activity (Supplementary Figure S1B,C). The mSense 5′UTR was sufficient to initiate translation and the translation commanded by the mAntisense 5′UTR was again more efficient (Supplementary Figure S1C). These results indicate that 5′UTRs can command translation only in cell culture and that translation commanded by the mAntisense SLE-1 5′UTR is more efficient, independent of the system used. 

As the mAntisense SLE-1 5′UTR can initiate translation using RRL, we asked if translation occurs in a cap-dependent manner. First, we tested the transcriptional activity of both the SLE-1 5′UTRs and mGlo in vitro with mRNAs without the 5′ cap structure (Figure 3C). We observed that as expected the mGlo reporter activity was lost, and no activity was observed for the mSense SLE-1 (Figure 3D). Surprisingly, we were able to detect translational activity when the mAntisense SLE-1 5′UTR was commanding translation of the FLuc reporter (Figure 3D). Then, we interrupted the eIF4E-eIF4G interaction cleaving eIF4G with FMDV L protease, thus targeting cap-dependent translation but not IRES-dependent translation [40,48,49,50]. The RRLs were treated with FMDV L protease, which efficiently cleaved eIF4G (Figure 3E). As expected for an mRNA whose translation depends on the cap structure, translation of the mGlo reporter was sensitive to the cleavage of eIF4G in a dose-dependent manner (Figure 3F). On the contrary, translation commanded by the mAntisense SLE-1 5′UTR was resistant to the inhibition of canonical translation by the FMDV L protease (Figure 3G). These results indicate that translation of *Mch*SLE-1 antisense mRNA initiates in a cap-independent manner, suggesting the possibility of IRES activity in the 5′AS mRNA of *Mch*SLE-1.

### 3.3. The IRES Activity of the MchSLE-1 5′UTR AS Requires a Nuclear Experience

**Figure 4:**
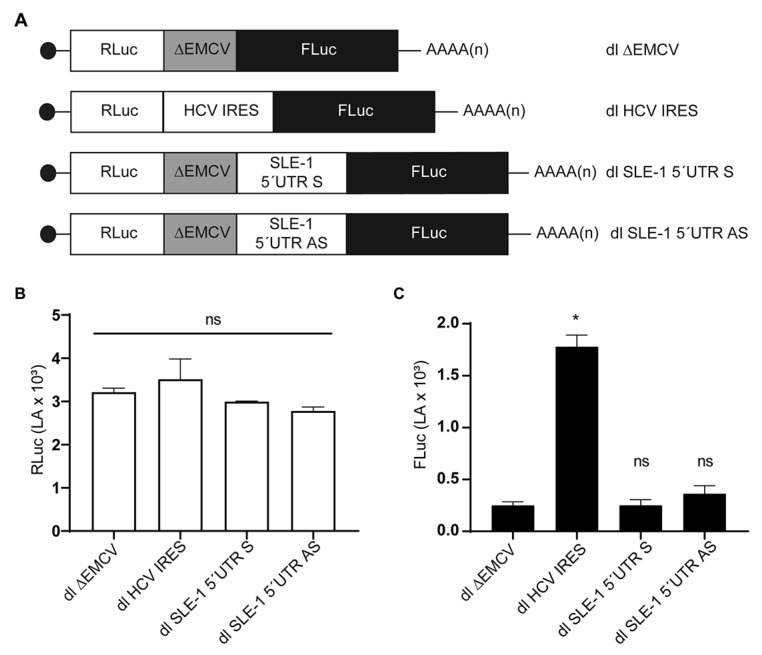
*Mch*SLE-1 5′ UTR S and AS are unable to drive translation of the second cistron when delivered as bicistronic mRNA into cells. The putative IRES activity of *Mch*SLE-1 5′UTR AS was tested by delivering dual-luciferase (dl) reporter mRNA into cells. (**A**) A schematic representation of the bicistronic mRNA reporter transfected into the HeLa cells. The black dot at the beginning of each scheme represents the 5′ cap. (**B**) The activity of the Renilla luciferase encoded on the first cistron is shown. (**C**) The activity of the Firefly luciferase, encoded on the second cistron, is shown. The values presented are the means of three independent experiments, each conducted in duplicate. A statistical analysis was performed by a one-way ANOVA, followed by a Tukey’s multiple test comparison. * = *p* < 0.05, and ns = no significant.

Our in vitro assays indicate that the *Mch*SLE-1 5′UTR AS can command translation independent of the 5′ cap structure (Figure 3G). It has been reported that both retroviruses and retrotransposons can mediate translation by the IRES located in their 5′UTRs [14,29,31]; therefore, we asked if the *Mch*SLE-1 5′UTR AS could initiate the translation via an IRES. To test this, we introduced the *Mch*SLE-1 5′UTRs (S and AS) into bicistronic reporters (Figure 4A). Bicistronic reporters or a dual (dl)-luciferase reporter are the most common method used to test IRES activity [51]. Here, the translation of the first cistron, RLuc in our case, occurs in a cap-dependent fashion, and the translation of the second cistron, FLuc in our case, will occur only if the intercistronic region has IRES activity [28,51]. An important control to prevent ribosomes that are translating the first cistron from passing to the second cistron is the insertion of the ∆EMCV sequence upstream of the intergenic region to inhibit ribosome reinitiation and readthrough [37,51]. 

To test if the IRES activity observed in the in vitro assays could also be observed in cells, we delivered the bicistronic mRNA reporter into the HeLa cells. As the positive control of the IRES activity, we used the dl HCV IRES (Figure 4A) [40,52,53], containing the Hepatitis C IRES in the intergenic region, while our negative control was dl ∆EMCV (Figure 4A) that does not have an intergenic region and does not present IRES activity [37,40,54,55]. We also included the dl SLE-1 5′UTR S along our dl SLE-1 5′UTR AS as the control, since our in vitro experiments showed no translation activity. The bicistronic mRNAs synthesized in vitro were capped and polyadenylated (Figure 4A) and then transfected into the HeLa cells. Six hours after, the transfection luciferase activity was measured. The reporter activity of the first cistron, RLuc, was equivalent, indicating that equivalent mRNA amounts were transfected and that RLuc activity occurred in a cap-dependent fashion from all the reporters (Figure 4B). Similar to what has been previously described [38,52,53], the reporter activity of the second cistron, FLuc, was barely detected from the dl ∆EMCV reporter and was high when the HCV IRES was used as the intergenic region. Furthermore, we did not observe IRES activity when either of the 5′UTRs of SLE-1 were used as the intergenic regions (Figure 4C).

**Figure 5:**
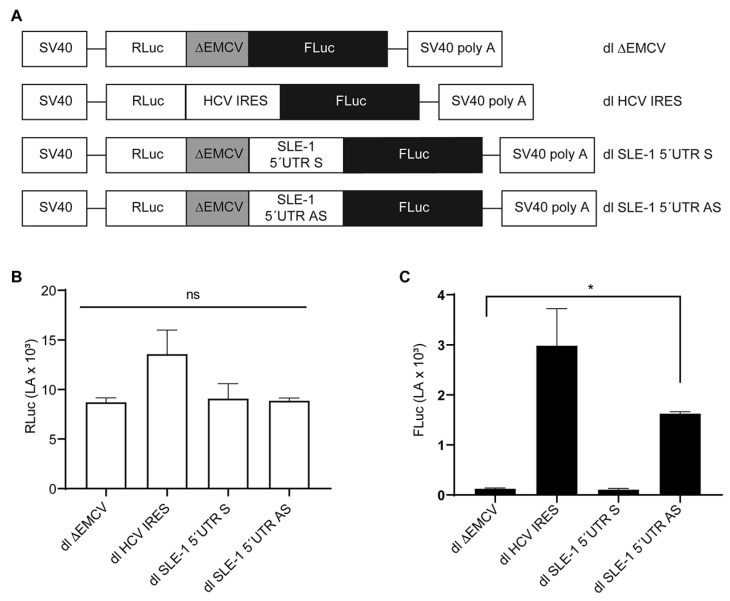
*Mch*SLE-1 5′ UTR AS initiates translation of the second cistron via an IRES. (**A**) A schematic representation of the plasmids encoding dl SLE-1 5′UTR S, dl SLE-1 5′UTR AS, dl ∆EMCV and dl HCV IRES transfected in HeLa cells. (**B,C**) At 24 h post transfection, the RLuc (**B**) and FLuc (**C**) activities were measured, and the results are presented as the luciferase activity (LA). The values shown are the means of three independent experiments, each conducted in duplicate. A statistical analysis was performed by a one-way ANOVA, followed by a Tukey’s multiple test comparison. * = *p* < 0.05, and ns = no significant.

The in vitro and in vivo analysis of the 5′UTR AS translation activity seemed to be contradictory; while the in vitro analysis suggested IRES activity, the in vivo assays showed no IRES activity when the bicistronic reporter was delivered to the cells as mRNA. It has been previously shown that the HIV-1 IRESs require a nuclear experience to be active, meaning that when the mRNA is transcribed in the nucleus, they present IRES activity, but if delivered directly to the cytoplasm, they either have weak or no IRES activity [56,57]. Therefore, we wondered if there was IRES activity on the 5′UTR AS when the bicistronic reporters are delivered as plasmids and mRNA synthesis occurs in the nucleus. For this, all the bicistronic reporters were cloned under the control of the SV40 promotor (Figure 5A). These plasmids were transfected in HeLa cells, and the 24 h post transfection luciferase activity was measured (Figure 5B,C). As expected, the reporter activity of the first cistron (RLuc) showed no significant differences between each bicistronic vector, indicating that RLuc synthesis occurs in a 5′cap-dependent manner and that an equivalent amount of each plasmid was transfected (Figure 5B). When the reporter activity of FLuc, encoded in the second cistron, was analyzed, we observed that the HCV IRES is significant and superior to the negative control but also that the 5′UTR AS region of *Mch*SLE-1 can command translation under these conditions (Figure 5C). This indicates that the 5′UTR AS region of *Mch*SLE-1 can internally recruit ribosomes in a bicistronic context, suggesting that the 5′UTR AS region of *Mch*SLE-1 exhibits IRES activity only when this mRNA is generated in the nucleus. 

**Figure 6:**
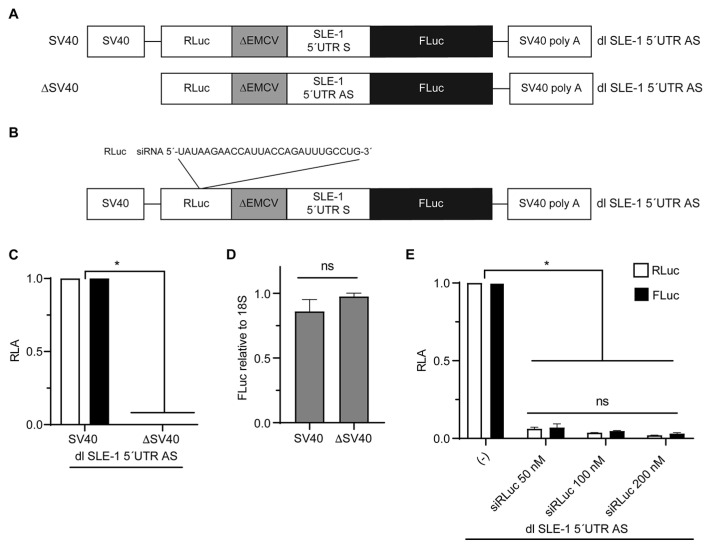
The synthesis of FLuc encoded in the bicistronic plasmid is exclusively attributed to the IRES activity of the *Mch*SLE-1 5′ UTR (AS). (**A**) A schematic representation of the dl SLE-1 5′UTR AS and promoterless ∆SV40 dl SLE-1 5′UTR AS plasmids transfected in HeLa cells. (**B**) A schematic representation of the dl SLE-1 5′UTR AS and the siRNA against the Renilla luciferase coding sequence. (**C**) HeLa cells were transfected with a dl SLE-1 5′UTR AS or ∆SV40 dl SLE-1 5′UTR AS bicistronic vector. The luciferase activity was measured 24 h post transfection. (**D**) As a control of homogenous transfection, the amounts of transfected DNA were determined by qPCR with primers directed to the Firefly luciferase in an aliquot of the sample used to measure the luciferase activity. The results are presented relative to the 18S gene amount. (**E**) The dl SLE-1 5′UTR AS was co-transfected along the RLuc siRNA (50, 100 and 150 nM) in the HeLa cells. The luciferase activity was measured 24 h post transfection. The results for the Renilla (white bars) and Firefly (black bars) luciferase are expressed as relative to the non-siRNA-treated cells. The means ± standard errors for three independent experiments, each performed in duplicate, are shown. A statistical analysis was performed using *t* tests. * = *p* < 0.05, and ns = no significant.

It has been described that when bicistronic reporters are transfected as plasmid DNA, artifactual IRES activity can be observed [51]. To rule out that the FLuc activity was due to artifactual results mediated, for example, by cryptic promoters or alternative splicing variants that could generate monocistronic FLuc mRNAs [51,58], we constructed a plasmid encoding the bicistronic mRNA but lacking the SV40 promoter (Figure 6A). This plasmid was transfected into HeLa cells and the luciferase activity was measured and compared with the plasmid containing the SV40 promoter. We did not observe reporter activity when the cells were transfected with the promoterless (∆SV40) plasmid (Figure 6C). To confirm that equivalent amounts of plasmid were transfected under both conditions, the amount of DNA was quantified by qPCR, detecting similar amounts of the luciferase DNA (Figure 6D). This indicated that FLuc synthesis in the dl SLE-1 5′UTR AS is not driven by a cryptic promoter. To rule out that the synthesis of FLuc was a consequence of alternative splicing, mediated by sequences in the 5′UTR or other sequences preceding the FLuc ORF, the dl SLE-1 5′UTR AS vector was co-transfected with increasing concentrations of an siRNA targeting the RLuc ORF (Figure 6B). In the presence of the siRNA, both the RLuc and FLuc activities decrease equivalently (Figure 6E), indicating that both reporter proteins are produced from the same mRNA, and there is no monocistronic mRNA for the FLuc. Therefore, no splicing events are interfering with the recorded activity of the FLuc. Together, these experiments indicate that the 5′UTR AS of *Mch*SLE-1 is capable of initiating translation independently of the 5′cap structure in a bicistronic context, exhibiting IRES activity. 

**Figure 7:**
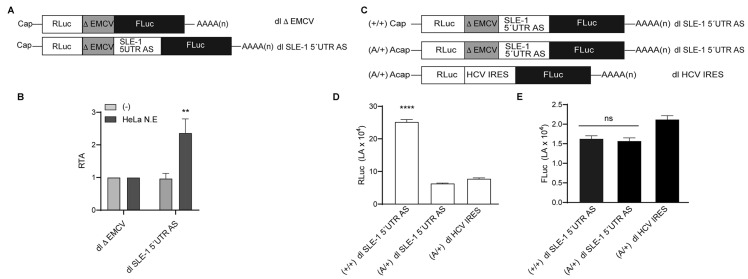
The *Mch*SLE-1 5′ UTR AS IRES is stimulated in vitro by nuclear extracts and has activity independent of the 5′-cap presence. (**A**) A schematic representation of the mRNAs incubated with HeLa cell nuclear (HeLa N. E.) extract before translation in RRL. (**B**) The mRNAs were treated (dark grey) or not (light grey) with 1 μg of HeLa N. E. for 10 min before in vitro translation in RRL. The relative translation activity (RTA) was calculated after the ratio FLuc/RLuc for each condition and was compared to dl ∆EMCV arbitrarily set to 1. (**C**) A schematic representation of the capped and polyadenylated (+/+) or Acap-modified and -polyadenylated (A/+) mRNAs used to test the IRES activity in vitro. (**D**) The activity of the Renilla luciferase encoded on the first cistron is shown. (**E**) The activity of the Firefly luciferase, encoded on the second cistron, is shown. The means ± standard errors for three independent experiments, each performed in duplicate, are shown. A statistical analysis was performed by one-way ANOVA. ** = *p* < 0.0012; **** = *p* < 0.0001, ns = no significant.

The above results show that the 5′UTR AS region of *Mch*SLE-1 has IRES activity when the bicistronic mRNAs are generated in the cell nucleus (Figure 5) and not when delivered directly to the cytoplasm (Figure 4). This suggests that this IRES requires nuclear proteins or a “nuclear experience”. As a first approach to test this, we returned to the in vitro RRL system and evaluated the translational activity of the bicistronic reporter as mRNA but adding HeLa cell nuclear extracts to the mRNA. For this assay, we used the dl ∆EMCV mRNA as the negative control and the dl SLE-1 5′UTR AS (Figure 7A) and calculated the relative translation activity (RTA), which is obtained by dividing the FLuc by the RLuc activity. We observed that the treatment of RNA with nuclear extracts before the in vitro translation significantly increases the translation activity of the IRES present in the dl SLE-1 5′UTR AS mRNA, compared to the untreated reaction, while no effect was observed for the dl ∆EMCV mRNA (Figure 7B). This result confirmed that a nuclear experience is required for the IRES activity present in the *Mch*SLE-1 5′UTR AS.

Although, in all our previous experiments, dl SLE-1 mRNAs and plasmids contain the ∆EMCV sequence that does not have IRES activity and blocks the readthrough from the first to the second cistron [37,40,54,55], the use of 5′-capped and non-capped mRNAs is also an important tool to dissect if the reporter activity of the second cistron is driven by the IRES and not by readthrough. We have adapted this tool and generated dl RNA that, in place of the 5′-cap, are modified by the ApppA (Acap), an analog that cannot be recognized by eIF4E; thus, it cannot initiate canonical translation [59,60]. For this assay, we included the dl SLE-1 5′UTR AS mRNA that was capped and polyadenylated (+/+), the Acap and polyadenylated version of this bicistronic reporter (A/+) and the dl HCV IRES with the Acap and polyadenylated version (A/+) as the positive control of the IRES activity (Figure 7C). When we analyze the reporter activity of the first cistron (RLuc) on the dl SLE-1 5′UTR AS mRNA, we observed, as expected, that the capped RNA has a strong activity and that this reporter activity is significantly decreased when the 5′-cap was replaced by Acap (Figure 7D). The dl HCV IRES RLuc activity was also low since it also has Acap (Figure 7D). However, when we analyze the reporter activity of the second cistron (Fluc), we observed that both Cap and Acap dl SLE-1 5′UTR AS RNAs have similar levels of activity, even comparable to the positive control dl HCV IRES (Figure 7E). This result confirms that the 5′UTR AS region of *Mch*SLE-1 has authentic IRES activity.

### 3.4. hnRNPK Is an IRES Trans-Activating Factor of MchSLE-1 IRES

Taking into consideration that retroviral mRNAs are naturally generated in the nucleus and that the activity of retroviral IRESs depends on IRES trans-activating factors (ITAFs) [14,61,62], we decided to evaluate the effect of heterogeneous nuclear ribonucleoprotein K (hnRNPK) on the activity of the *Mch*SLE-1 IRES. hnRNPK is an ITAF of the picornaviral IRES of FMDV [63] and has been recently described as a retroviral IRES ITAF [41]. 

**Figure 8:**
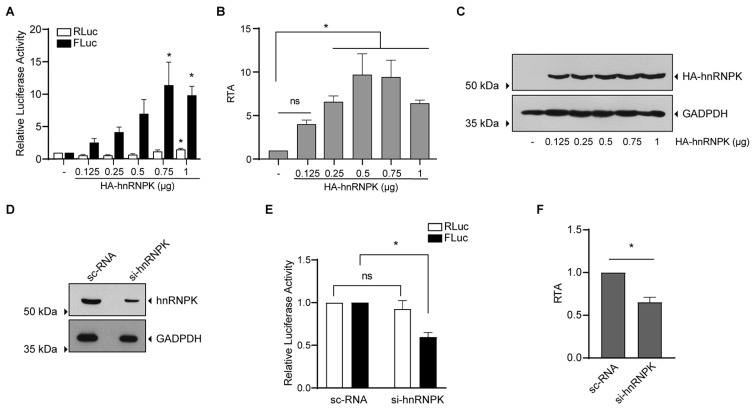
The heterogeneous nuclear ribonucleoprotein K (hnRNPK) is a positive ITAF for the *Mch*SLE-1 IRES. (**A**) HeLa cells were co-transfected with dl SLE-1 5′UTR AS and increasing amounts of the plasmid encoding HA-hnRNPK. The luciferase activity was measured 24 h post transfection. The RLuc (white bars) and FLuc (black bars) relative to the dl SLE-1 5′UTR AS alone are shown as the relative luciferase activity (RLA). (**B**) The relative translation activity (RTA) was calculated after the ratio FLuc/RLuc for each condition and was compared to the dl SLE-1 5′UTR AS arbitrarily set to 1. (**C**) A Western blot assay of the cell lysates using anti-HA antibody to detect HA-hnRNPK. GAPDH detection was used as the loading control. (**D**) A Western blot assay of the cells treated with sc-RNA or siRNA directed against the hnRNPK (si-hnRNPK), anti-hnRNPK or anti-GAPDH antibodies used. For (**C**,**D**) the migration of the proteins is depicted on the right-hand side and the migration of the molecular marker is depicted on the left-hand side. (**E**) The HeLa cells were co-transfected with dl SLE-1 5′UTR AS and sc-RNA or si-hnRNPK RNAs. The luciferase activity was measured 24 h post transfection. The RLuc (white bars) and Fluc (black bars) relative to the dl SLE-1 5′UTR AS sc-RNA is shown as the RLA. (**F**) The RTA was calculated. The means ± standard errors for three independent experiments, each performed in duplicate, are shown. A statistical analysis was performed by one-way ANOVA. * = *p* < 0.05, and ns = no significant.

When the dl SLE-1 5′UTR AS was co-transfected with increasing amounts of a plasmid encoding HA-hnRNPK, we observed that the cap-dependent translation of the first cistron (RLuc) was not affected (Figure 8A, white bars), while the IRES-dependent translation of the second cistron (FLuc) was increased in a dose-dependent manner (Figure 8A, black bars). The positive effect of hnRNPK over *Mch*SLE-1 IRES activity is better appreciated when the RTA (FLuc/RLuc) was calculated. We observed a significant increase in the IRES activity starting at 0.25 µg of the HA-hnRNPK-encoding plasmid (Figure 8B). The overexpression of HA-hnRNPK was confirmed by Western blot (Figure 8C). To confirm that hnRNPK is a positive ITAF for *Mch*SLE-1 IRES, and that the results described above were not an artifact of hnRNPK overexpression, we silenced the endogenous protein using siRNA. By transfecting 20 nM of an siRNA targeting the 3′UTR of hnRNPK mRNA, we observed a clear reduction in the hnRNPK protein compared to the sc-RNA (Figure 8D). This protein reduction did not affect the cap-dependent translation of the first cistron RLuc (Figure 8E, white bars) but was enough to reduce the IRES-dependent translation of the second cistron FLuc by 50% (Figure 8E, black bars). This is also clear in the RTA analysis (Figure 8F). Taken together, the gain and loss of function experiments indicate that as for the HIV-1 IRES [41], hnRNPK is a positive ITAF for the *Mch*SLE-1 IRES. 

### 3.5. The AS ORF of MchSLE-1 Can Be Expressed as Protein in Cell Culture

**Figure 9:**
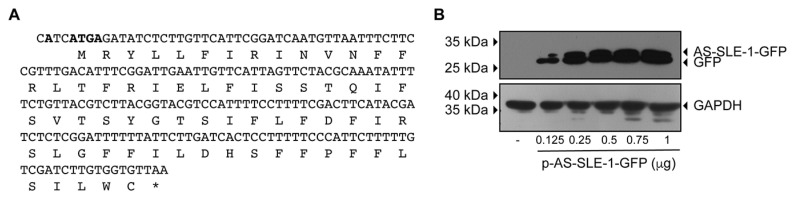
The AS ORF of *Mch*SLE-1 can be expressed as protein in the cell culture. The AS-SLE-1 coding sequence was cloned into the pEFGP-N1 vector to generate the plasmid p-AS-SLE-1-GFP that allows for expression of the AS-SLE-1-GFP fusion protein in the cell culture. (**A**) The nucleotide and amino acid sequences of the AS-SLE-1 ORF are presented; in bold, the KOZAK context found in the *Mch*SLE-1 genome is depicted. This context was conserved during the cloning and the stop codon, depicted here with *, was eliminated. (**B**) HeLa cells were transfected (or not), with increasing amounts of p-AS-SLE-1-GFP. Twenty-four hours after transfection, the cells were lysed and Western blot assays using anti-GFP or anti-GAPDH antibodies were performed. A representative Western blot of three independent experiments is shown. The migration of the proteins is depicted on the right-hand side, and the migration of the molecular marker is depicted on the left-hand side.

Taking into consideration that *Mch*SLE-1 produces transcripts in both senses, the AS transcripts contain an ORF of 67 amino acids (Figure 9A) that we named AS-SLE-1 ORF and the 5′UTR of this transcript can command translation by its IRES activity (Figure 3, Figure 5 and Figure 7), we asked if a protein is expressed from this sequence. As we do not possess antibodies for the putative protein encoded in the AS-SLE-1 ORF, we decided to test the ability of the putative protein to be expressed in a heterologous system. For this, we cloned the AS-SLE-1 ORF coding sequence in fusion to GFP, replacing the natural ATCATGA with AGCATGA during the cloning. In this construct, the translation of the fusion protein should start with the initiation codon of the AS-SLE1 ORF to generate the protein AS-SLE-1-GFP, with GFP at the C-terminal of AS-SLE-1. Upon transfection of increasing doses of the p-AS-SLE-1-GFP plasmid, we observed that the anti-GFP antibody recognizes a protein of a higher molecular weight than GFP. We also observed the expression of GFP alone. It is not uncommon that GFP fusion proteins also express GFP that could be a cleavage product of the fusion, or that can be expressed on its own since its initiation context was not altered by the cloning. This result suggests that in the natural context, it could be possible that the AS-SLE-1 ORF is indeed expressed as protein. Further experiments will be required to confirm this. 

## 4. Discussion

In this article, we show that *Mch*SLE-1, an LTR-retrotransposon previously described in the genome of the Chilean blue mussel *M. chilensis*, is transcribed from both strands (Figure 2) and that both S and AS 5′UTRs can potentially command translation of these transcripts. When translation was evaluated in vitro, S 5′UTR-mediated translation was not detected (Figure 3B), while AS 5′UTR can initiate protein synthesis, even independently of the 5′cap structure (Figure 3D,E). This occurs via an IRES that requires nuclear experience (Figure 5, Figure 7 and Figure 8). 

To evaluate the ability of these 5′UTRs to drive translation, we used rabbit reticulocyte lysates (RRL), a conventional tool for studies of eukaryotic translation initiation in vitro [59]. The S 5′UTR should command the translation of the transposase required for mobilization of the LTR-retrotransposon. The lack of activity for the S 5′UTR during in vitro assays can be due to the need of a nuclear experience of the mRNA for non-conventional cap-dependent translation. Non-canonical mechanisms of cap-dependent translation initiation have been described. Certain mRNAs that are translated in a cap-dependent manner, referred to as TOP RNAs, must be generated in the nucleus to become active [64]. TOP RNAs contain in their 5′ terminal oligopyrimidine (TOP) sequences that allow for translation in specialized conditions [65]. Other mRNAs initiate cap-dependent translation via the Cap-binding complex (CBC) and the nuclear CBC-interacting proteins [66]. This is the case of the cap-dependent translation mechanism of HIV-1 full-length RNA that uses CBP80, a component of CBC, to ensure protein synthesis during the HIV-1 cycle [67]. To test if S 5′UTR translation activity required a nuclear experience, we also tested the monocistronic *Mch*SLE-1 reporters in cells. For this, we delivered plasmids that allow for the nuclear synthesis of the monocistronic reporters (Supplementary Figure S1). We were able to detect the translation activity of the S 5′UTR, although this was about four folds lower than the AS 5′UTR activity (Supplementary Figure S1C). Therefore, it is possible that S 5′UTR is a TOP RNA or that it uses a nuclear factor to mediate cap-dependent translation. Further experiments are required to test these hypotheses.

The differences we observed in the translation activity of the *Mch*SLE-1 S 5′UTR in the RRL versus HeLa cells and between the S and AS 5′UTRs could also be attributed to the structure of these RNAs. We modeled the structure of both mRNA in silico (Supplementary Figure S2) and found important differences. The *Mch*SLE-1 S 5′UTR model shows that it has high probability of base pairing and low entropy (Supplementary Figure S2A), which suggests a highly stable RNA structure. On the contrary, the *Mch*SLE-1 AS 5′UTR model shows a low base pairing probability and high entropy, suggesting a dynamic structure that is easier to open after recruiting the translation machinery and to be scanned to initiate translation. These models do not consider the influence of proteins that can bind these RNAs but help explain our results. Here, we showed that the *Mch*SLE-1 S 5′UTR is unable to command translation in vitro (Figure 3B), but when the RNA is transcribed in the cell nucleus, transcription does occur (Supplementary Figure S1). The mRNA secondary structures must be solved during translation by RNA helicases, a step that strongly influences the translation of eukaryotic mRNAs [68]. Eukaryotic RNA helicases participate in several processes of RNA metabolism, such as transcription, splicing, nuclear export and translation [68,69,70]. It is possible then to envision that the sense mRNA of the LTR-retrotransposon *Mch*SLE-1 could require the assistance of RNA helicases found in the cell nucleus to be translated that will help untangle the highly stable secondary structure predicted.

In the in vitro system, the *Mch*SLE-1 AS 5′UTR drives translation in a cap-dependent manner and in a cap-independent manner, since the translation activity partially persists when the mRNA has no 5′cap or is modified by Acap (Figure 3D and Figure 7E) and is resistant to FMDV L protease treatment (Figure 3G). This protease cleaves the N-terminal end of eIF4G [50], preventing the eIF4E-eIF4G binding required for canonical cap-dependent translation. This observation led us to propose that the *Mch*SLE-1 AS 5′UTR might have IRES activity. To test this, we used mRNAs and bicistronic vectors [51] that were delivered in HeLa cells. We show that the *Mch*SLE-1 AS 5′UTR exhibits IRES activity (SLE-1 IRES) and requires being transcribed in the nucleus to become active (Figure 4 and Figure 5). The activity of the SLE-1 IRES behaves similar to the HIV-1 IRES, for whom it has been proposed that it requires a nuclear experience [57,71]. The nuclear experience is associated with the binding of factors that facilitate nuclear export to the cytoplasm for subsequent translation [14,72]. It has been described that the nuclear proteins hnRNPL and nucleolin bind LINE-1 RNA and modulate the activity of the retrotransposon via the IRES regulation of its translation [73]. In our case, the requirement of this nuclear experience by the *Mch*SLE-1 IRES led us to evaluate the effect of nucleus-cytoplasm shuttling proteins like hnRNPK on *Mch*SLE-1 IRES activity. hnRNPK has been described as an IRES trans-acting factor (ITAF) for picornaviruses [74,75], flaviviruses [63,76] and retroviruses [41]. In this study, we demonstrate that hnRNPK positively modulates the activity of the SLE-1 IRES (Figure 8). Further experiments are necessary to determine whether this stimulation occurs through the direct interaction of hnRNPK with the SLE-1 IRES or through protein–protein–RNA interactions. Additionally, it has been proposed that retroviral IRES elements depend on several ITAFs, where a combination of RNA-binding proteins and their post-translational modifications modulate the activity of an IRES, the retroviral IRESome model [14]. It would be interesting, in the future, to dissect the IRESome of LTR-retrotransposons.

The use of an IRES by cellular or retroviral RNAs occurs mainly during stress, apoptosis, cell division or cancer, events in which canonical translation initiation is inhibited [77,78]. This led us to think that *Mch*SLE-1 AS transcripts, if indeed translated in the mussel, could be responding to some kind of stress or might have a function during transposition activity. Antisense transcripts have different functions in the retrovirus and retrotransposons pathogenesis and replication cycle [12]. ASP-L, a 2.6 kb antisense transcript of HIV-1, exhibits a dominant suppressor activity and can lock an integrated provirus [8], while ORF0 of the LINE-1 retrotransposon encodes a small protein that enhances the mobility of this element and participates in host splicing events [10]. As a first approach to test if *Mch*SLE-1 A and AS transcripts could be translated, we expressed the AS-SLE-1 ORF as a fusion protein in HeLa cells (Figure 9); this suggest that these transcripts could be translated in the mussel. Further experiments are needed to determine if indeed both transcripts are translated in animals and if the putative antisense protein is regulating the expression of the transposase encoded by the LTR-retrotransposon as ASP-L and ORF0 do. Also, further experiments will be needed to determine if both transcripts are derived from the same locus or if any of the five *Mch*SLE-1 loci described in the *M. chilensis* genome [36] are transcribing in the antisense strand to regulate expression of the other ones. 

All the experiments presented here were performed in vitro or in HeLa cells; this was because to our knowledge, there is no cell line derived from mollusks that could be used to test the translation of the reporter mRNA used in this study. It is possible to culture mussel hemocytes for a few days [79], but there is the issue of transfectability and possible variability among individuals, since many donors must be combined to have an adequate number of cells to perform this kind of experiment. The use of HeLa cells to study retroviral RNA translation has been widely validated [41,57,72,80]. Therefore, we considered them an appropriate model system to study the translation initiation of an LTR-RT such as *Mch*SLE-1, but certainly it would be ideal to have mollusk cell lines were we to study mollusk LTR-RT. 

To our knowledge, this is the first evidence of an IRES in an antisense RNA within a long terminal repeat retrotransposon in mollusks and the first description of hnRNPK as an ITAF for an LTR-retrotransposon.

## Data Availability

All data are available upon request.

## Supplementary Materials

The following supporting information can be downloaded at: https://www.mdpi.com/article/10.3390/v16030403/s1, Supplementary Figure S1: MchSLE-1 5′UTR S initiate translation in cells when is transcribed in the nucleus. Supplementary Figure S2: Secondary structure prediction of *Mch*SLE-1 S and AS 5′UTRs.
