# Supplementary material for: The Mytilus chilensis Steamer-like Element-1 Retrotransposon Antisense mRNA Harbors an Internal Ribosome Entry Site That Is Modulated by hnRNPK
Source: Viruses. 2024 Mar 5;16(3):403. doi: 10.3390/v16030403 (PMC10974842; doi:10.3390/v16030403)
Supplement: Supplementary file 1 [file viruses-16-00403-s001.zip › viruses-2863332-supplementary.pdf]

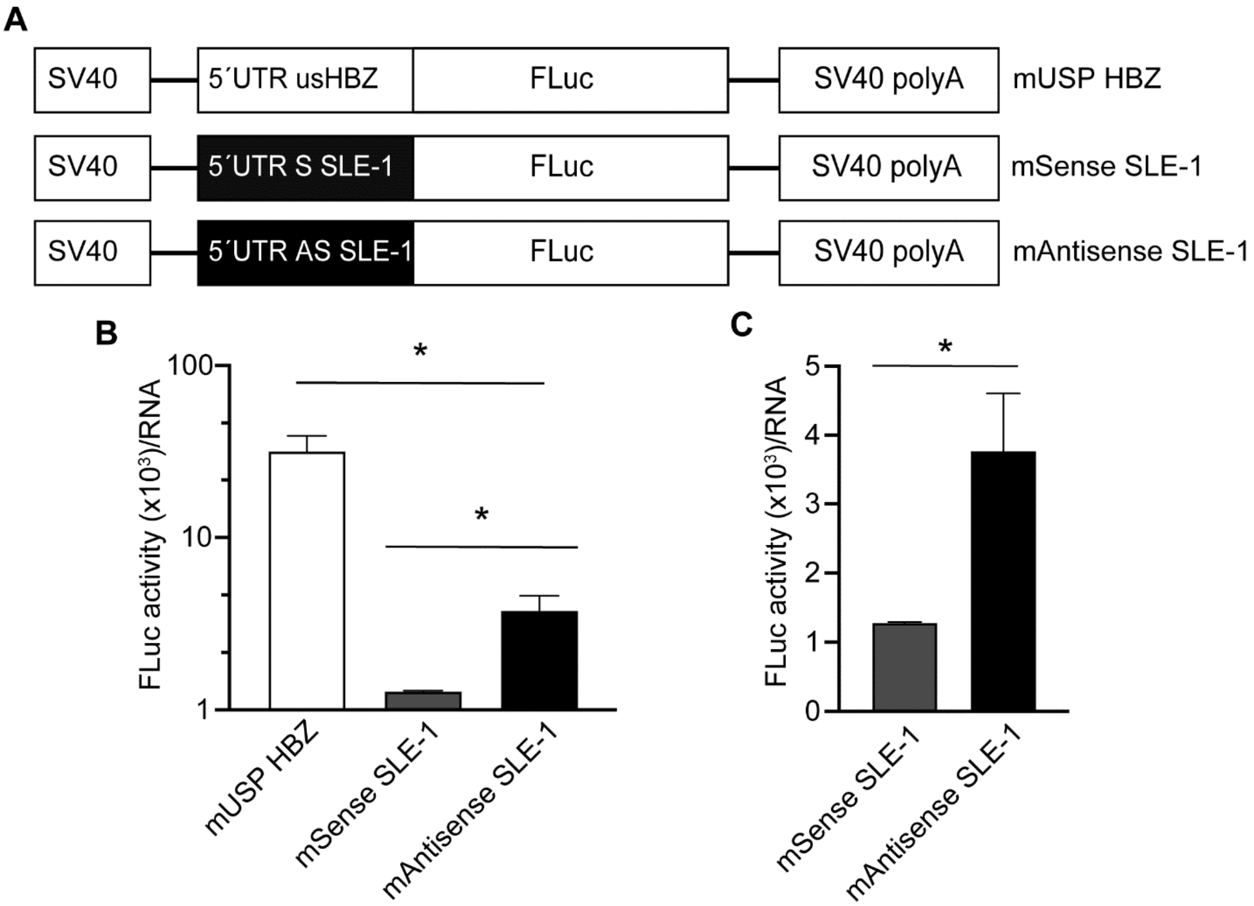

Scheme 1. MchSLE-1 5´UTR S initiate translation in cells when is transcribed in the nucleus. The capacity of monocistronic mRNAs to translate in cell culture was tested. **A.** Schematic representation of the plasmids encoding mUSP HBZ, mSense SLE-1 and mAntisense SLE-1 transfected in HeLa cells. **B.** Twenty-four hours after transfection FLuc activity was measured, results are presented as luciferase activity, normalized by the 18S RNA quantity measures by qPCR. The values shown are means of three independent experiments, each conducted in duplicate. **C.** Same data shown in B, without the activity of the positive cap-dependent translation, for a better appreciation of the mSense SLE-1 and mAntisense SLE-1 reporters activity. Statistical analysis was performed by t test. *=$p<0.05$.

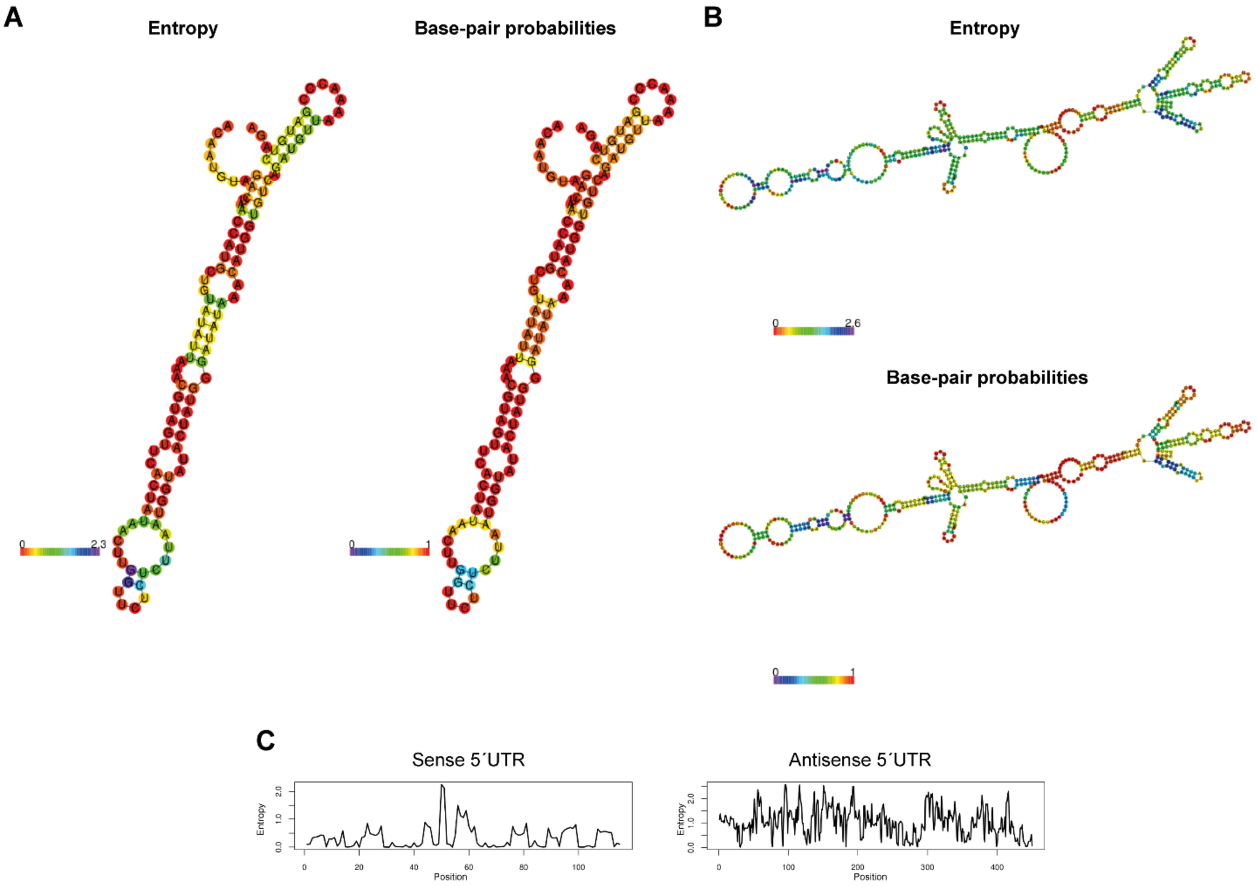

Scheme 2. Secondary structure prediction of *Mch*SLE-1 S and AS 5´UTRs. The secondary structures of the *Mch*SLE-1 S and AS 5´UTRs were predicted using RNAfold 2.6.3. **A and B.** Model base on positional entropy (left hand side) and base-pair probability (right hand-side) of the Sense (**A**) and Antisense (**B**) 5´UTR regions. The scale indicated the probability for each model as color intensity. **C.** The positional entropy for each nucleotide position is shown .
